# An Equine Herpesvirus Type 1 (EHV-1) Expressing VP2 and VP5 of Serotype 8 Bluetongue Virus (BTV-8) Induces Protection in a Murine Infection Model

**DOI:** 10.1371/journal.pone.0034425

**Published:** 2012-04-12

**Authors:** Guanggang Ma, Michael Eschbaumer, Abdelrahman Said, Bernd Hoffmann, Martin Beer, Nikolaus Osterrieder

**Affiliations:** 1 Institut für Virologie, Freie Universität Berlin, Berlin, Germany; 2 Institut für Virusdiagnostik, Friedrich-Loeffler-Institut, Greifswald-Insel Riems, Germany; University of Iowa, United States of America

## Abstract

Bluetongue virus (BTV) can infect most species of domestic and wild ruminants causing substantial morbidity and mortality and, consequently, high economic losses. In 2006, an epizootic of BTV serotype 8 (BTV-8) started in northern Europe that caused significant disease in cattle and sheep before comprehensive vaccination was introduced two years later. Here, we evaluate the potential of equine herpesvirus type 1 (EHV-1), an alphaherpesvirus, as a novel vectored DIVA (differentiating infected from vaccinated animals) vaccine expressing VP2 of BTV-8 alone or in combination with VP5. The EHV-1 recombinant viruses stably expressed the transgenes and grew with kinetics that were identical to those of parental virus *in vitro*. After immunization of mice, a BTV-8-specific neutralizing antibody response was elicited. In a challenge experiment using a lethal dose of BTV-8, 100% of interferon-receptor-deficient (IFNAR^−/−^) mice vaccinated with the recombinant EHV-1 carrying both VP2 and VP5, but not VP2 alone, survived. VP7 was not included in the vectored vaccines and was successfully used as a DIVA marker. In summary, we show that EHV-1 expressing BTV-8 VP2 and VP5 is capable of eliciting a protective immune response that is distinguishable from that after infection and as such may be an alternative for BTV vaccination strategies in which DIVA compatibility is of importance.

## Introduction

Bluetongue virus (BTV), the prototype of the genus *Orbivirus* within the family *Reoviridae*, is the causative agent of bluetongue disease in many species of domestic ruminants, especially sheep. The virus is highly infectious but not contagious; it is transmitted to ruminants by biting midges belonging to the genus *Culicoides* [1]. BTV has a genome composed of 10 segments of double-stranded RNA that encode for 7 structural proteins (VP1-VP7) and 5 non-structural proteins (NS1, NS2, NS3/3a, NS4) [2]. Of these, VP2 and VP5 are the major structural proteins forming the outer capsid of the virus particle. VP2 is the main determinant of BTV serotype and is responsible for receptor binding, hemagglutination, and induction of serotype-specific neutralizing antibodies, while VP5 influences virus neutralization through its conformational interaction with VP2 [3], [4]. VP7 and VP3 are the major core proteins and play important roles with respect to the structural integrity of virions [3]. At present, 26 distinct serotypes of BTV (BTV-1 to -26) are recognized, between which there is only little cross-protection, a fact that complicates vaccination strategies [3], [5], [6].

BTV was thought to circulate only in tropical and sub-tropical regions; however, an unusual epizootic of BTV serotype 8 (BTV-8) started in central and northern Europe in 2006, affecting both sheep and cattle. During the following years, BTV-8 has spread rapidly throughout Central Europe and caused massive economic losses [7], [8]. For safety reasons, only inactivated whole virus vaccines against several serotypes of BTV, including serotype 8, are now commercially available in Europe. The vaccines are highly efficacious in reducing clinical disease and BTV circulation [7]. Preparations of structurally intact BTV virions induce a broad immune response to virtually all BTV structural proteins and, in some cases, even non-structural proteins dependent on the production system [9]. Widespread use of such vaccines, therefore, confounds serological diagnosis and surveillance. The inability to differentiate between infected and vaccinated animals is of concern particularly in cattle, which are usually asymptomatic after BTV infection but can still spread the virus [10]. One possible approach to a vaccination strategy that allows the differentiation of infected from vaccinated animals (DIVA) is the use of recombinant vaccines expressing only a subset of BTV proteins. Proteins not included in the vaccine can then be used as negative markers. Promising results were reported using diverse poxviruses as live vectors for delivery of BTV antigens, mainly VP2, VP5 and VP7 [11], [12], [13], [14].

This study presents a different approach, using equine herpesvirus type 1 (EHV-1) as the delivery vector. EHV-1, a member of the genus *Varicellovirus* in the subfamily *Alphaherpesvirinae* [15], is endemic in many horse populations and causes mild to severe clinical disease that includes rhinopneumonitis, abortion and neurological disorders [16]. EHV-1 has a double-stranded DNA genome 150 kbp in length, with numerous nonessential genes that allow insertion of foreign sequences. The potential of EHV-1 as a universal immunization vector is highlighted by its high efficiency in delivering foreign genes in a wide variety of cells and the lack of anti-vector immunity in non-equine hosts [17], [18]. EHV-1 strain RacH, attenuated by continuous passaging on primary swine kidney cells, is currently used as a modified live vaccine (MLV) against EHV-1 infection in the US and Europe and has an excellent safety record [19]. This vaccine strain has been established as an infectious bacterial artificial chromosome (BAC), which makes manipulation of the virus genome easily accessible [20]. Based on the RacH strain, live-vectored vaccines have been developed against various viruses, which were shown to induce both humoral and cellular immune responses and provide protection in vaccinated animals, including mice, dogs and cattle [19], [21], [22], [23], [24].

Lately, interferon a/β receptor-deficient (IFNAR^−/−^) mice have been established as a novel small animal model of BTV infection [25]. IFNAR^−/−^ mice are impaired in their innate immune responses [26] and were shown to be highly susceptible to BTV infection, but they can be completely protected by vaccination, making them an ideal tool for BTV vaccine research [25].

Here, we describe the construction and evaluation of two EHV-1 RacH-based recombinant vaccines against BTV-8 expressing the immunodominant outer capsid protein VP2 alone or in combination with VP5. We show that both recombinant EHV-1 mutants stably express the transgenes and induce a BTV-8-specific neutralizing antibody response. In the IFNAR^−/−^ mouse model, VP2 alone was unable to protect mice against BTV-8 challenge; however, substantial protection was observed when VP2 and VP5 were used in combination. VP7 was not included in the recombinant vaccines and was used as a DIVA marker.

## Results

### Construction of recombinant viruses

**Figure 1:**
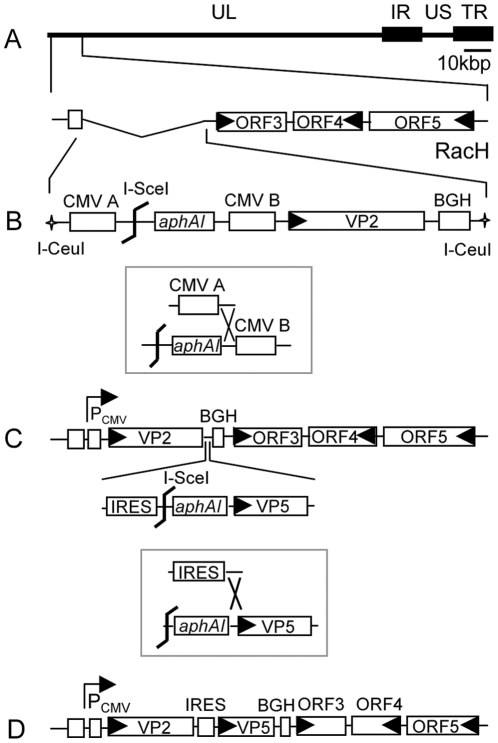
Schematic illustration of the construction strategies of the recombinant viruses. (A) The organization of the left terminus of the EHV-1 RacH genome showing that ORF1 and ORF2 are absent. UL: unique long; US: unique short; IR: internal repeat; TR: terminal repeat. (B) A fragment released from transfer plasmid pEP-VP2 by *I-Ceu*I digestion was used to recombine with RacH genome, resulting in an intermediate kanamycin (*aphAI* cassette)-resistant BAC clone. After *I-Sce*I digestion, kanamycin was removed in the following step of *en passant* mutagenesis (in box) to generate VP2-expressing virus. (C) With another round of *en passant* mutagenesis, VP5 gene with an IRES sequence upstream were inserted in between VP2 and BGH polyA, and a final construct expressing both VP2 and VP5 (D) was generated.

EHV-1 ORF1 and ORF2 have been shown to be dispensable for virus growth, are absent in the vaccine strain RacH [19], [22], [27], [28] and were chosen as the target region for transgene insertion. To avoid potential recombination of the CMV promoter upstream of *egfp* in mini-F sequences with the CMV promoter controlling transgene expression, parental pRacH1 was modified such that the CMV promoter upstream of *egfp* was replaced with an EF-1α promoter using two-step Red mutagenesis. Based on the modified pH1_EF1 BAC, a BTV-8 VP2 expression cassette was inserted in the ORF1/2 deletion region, resulting in pH1_EF1_VP2. In a next step, the BTV-8 VP5 gene with an upstream internal ribosome entry site (IRES) was inserted downstream of VP2, resulting in pH1_EF1_VP2_5 (Fig. 1). The intervening IRES sequence serves as a ribosome-binding site for the internal initiation of translation in a cap-independent fashion [29]. VP2 and VP5 were separated by the IRES sequence such that the two genes could be co-expressed as a single transcriptional unit under the control of the common upstream HCMV IE promoter. The correct genotype of all mutant BACs was confirmed by RFLP analysis using *Bam*HI or *Hin*dIII digestion. Upon transfection of BAC DNA into RK13 cells, the recombinant viruses, rRacH1_EF1, rH_EF1_VP2 and rH_EF1_VP2_5 were reconstituted. The expression of gp2 was repaired in the viruses by co-transfection of RK13 cells with viral DNA and plasmid DNA containing full-length gene 71, and the final mutants, rRacH1, rH_VP2 and rH_VP2_5 were generated. The VP2 and VP5 genes were sequenced in the recombinant viruses, which ensured that no mutations occurred during the recombination processes (data not shown).

### Transgene expression and *in vitro* growth properties of the recombinant viruses

**Figure 2:**
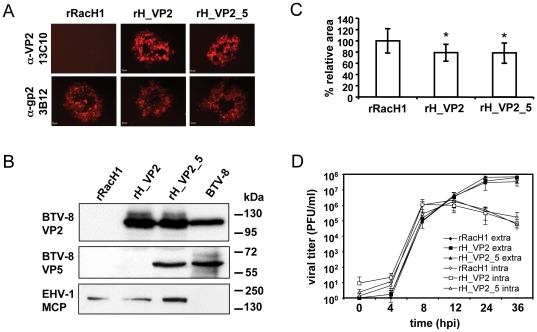
Expression of the transgenes and *in vitro* growth properties. (A) RK13 cells were infected with parental rRacH1, rH_VP2 or rH_VP2_5 at an m.o.i of 0.0001. Two days post infection, cells were fixed and incubated with anti-VP2 mAb 13C10 or anti-EHV-1 gp2 mAb 3B12, followed by Alexa Fluor 568-conjugated goat anti-mouse IgG. Fluorescence signal was inspected under the inverted fluorescence microscope. Bar indicates 50 µm. (B) Cell lysates infected by rRacH1, rH_VP2, rH_VP2_5 or BTV-8 were separated by 10% SDS-PAGE and analysed by Western blot. Expression of VP2 and VP5 was detected using primary antibody 13C10 and sheep anti-BTV-8 hyperimmune sera, respectively. EHV-1 MCP was used as a control and detected with mAb 3G4. (C) RK13 cells were infected by the individual virus at an m.o.i of 0.0001 and overlaid. Three days post infection, plaques were photographed and the areas were measured. For each virus, at least 50 plaques were measured. The relative plaque area was compared to that of rRacH, which was set as 100%. * *P*<0.001. (D) The single-step growth kinetics of these viruses was analysed. RK13 cells were infected by the viruses at an m.o.i of 5. Extracellular and intracellular virus titres were determined at the indicated time points.

To determine whether the recombinant viruses expressed VP2 and VP5, IFA and western blot analyses were performed. Using VP2 mAb 13C10, a specific signal could be detected in cells infected with either rH_VP2 or rH_VP2_5, but not in cells infected with the parental rRacH1 virus. As a control, EHV-1 gp2 expression could be detected in cells infected by either of these viruses (Fig. 2A). Because a specific mAb against VP5 was not available, the expression of VP5 could not be tested using IFA. In western blot analyses using sheep anti-BTV-8 hyperimmune sera, a specific band with a size of around 60 kDa could be detected in lysates of rH_VP2_VP5-infected RK13 cells and BTV-8-infected Vero cells but not in those from rH_VP2- or rRacH1-infected cells (Fig. 2B). We concluded from the specificity of detection and the size of the reactive band that VP5 was expressed from rH_VP2_VP5 but not from the other two viruses. Consistent with the IFA results, VP2, with a predicted mass of 106kDa, could be detected in RK13 cells infected with rH_VP2 or rH_VP2_VP5, but not in those infected with rRacH1 (Fig. 2B). Both VP2 and VP5 recombinant proteins were shown to co-migrate with wild-type virus proteins from Vero cells infected with BTV-8 (Fig. 2B). Expression of VP2 and VP5 remained stable during continuous virus passage in RK13 cells as tested by both IFA and western blotting after 10 passages.

Knowing that the transgenes were stably expressed, the *in vitro* growth properties of the recombinant viruses were compared with those of parental virus rRacH1. The ability of the viruses to spread from cell to cell was determined by comparison of relative plaque areas. With the insertion of the VP2 expression cassette or VP2 and VP5 in combination, the recombinant viruses displayed reduced plaque areas that were about 20% smaller than those formed by rRacH1 when measured on day 3 p.i. (*P*<0.05) (Fig. 2C). To further examine the replication properties of the recombinant viruses, single-step growth kinetics were determined. We could demonstrate that both extracellular and intracellular titers of rH_VP2 and rH_VP2_5 were comparable with those of parental rRacH1 during a 36 h period (Fig. 2D). The results revealed that rH_VP2 and rH_VP2_5 were only slightly impaired in terms of cell-to-cell spread, but that infectious virus production was not affected by the insertion and expression of the BTV-8 antigens.

### Vaccination with rH_VP2 and rH_VP2_5 induces a neutralizing antibody response against BTV-8

**Figure 3:**
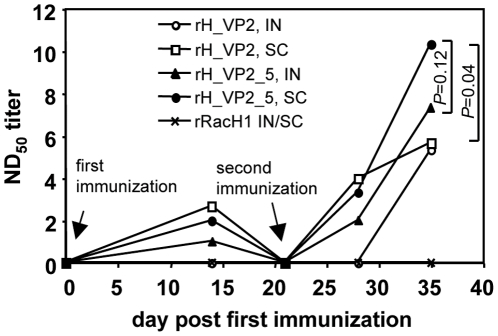
Neutralising antibody response was induced by the recombinant viruses. Three-week old female Balb/c mice were prime/booster immunised with rRacH1, rH_VP2 or rH_VP2_5. At the indicated days (0, 14, 21, 28, 35), mice were bled and the antibody was examined using standard serum neutralisation test. IN: intranasal; SC: subcutaneous.

To determine if the recombinant viruses can induce neutralizing antibody responses against BTV-8, pilot vaccination of Balb/c mice was conducted. The mice were immunized twice by the IN or SC route with rH_VP2, rH_VP2_5 or rRacH1, and the neutralizing antibody titers were measured using a standard serum neutralization test (SNT). Neutralizing antibodies were detected in mice inoculated with either rH_VP2 or rH_VP2_5, but not in mice that had been inoculated with rRacH1 (Fig. 3). While neutralizing antibodies were detected as early as day 14 in mice immunized by the SC route with rH_VP2 or in mice vaccinated with rH_VP2_5 by either route, neutralizing activity was detectable only from day 35 (2 weeks after booster immunization) in mice receiving rH_VP2 IN. For rH_VP2_5, the antibody titer after immunization by the SC route was also higher, albeit not significantly, than that after IN immunization, with a maximum difference on day 35 (*P* = 0.12, T-test). When the two recombinant viruses were compared, the neutralizing antibody response induced by rH_VP2_5, although slightly weaker at day 14 and day 28 than that induced by rH_VP2 given by the SC route, reached significantly higher levels on day 35 (*P* = 0.04, T-test) (Fig. 3). On the basis of these results, we concluded that both rH_VP2 and rH_VP2_5 were able to induce a specific anti-BTV-8 antibody response *in vivo* and that rH_VP2_5 was more effective than rH_VP2 with respect to inducing a neutralizing antibody response.

### rH_VP2_5, but not rH_VP2, protects mice against BTV-8 challenge

**Figure 4:**
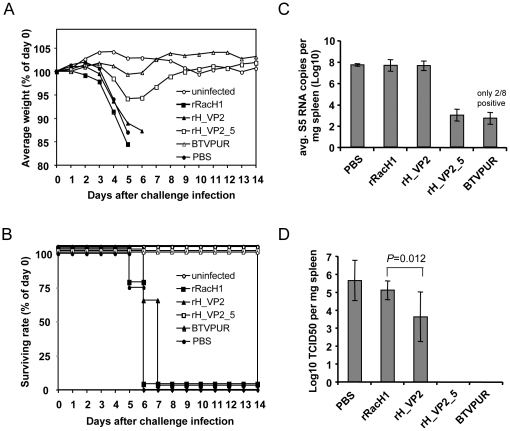
Protective efficacy of the recombinant vaccines against BTV-8 challenge in IFNAR^−/−^ mice. (A) Average group weights after challenge. (B) Survival rate of IFNAR^−/−^ mice after BTV-8 challenge infection. (C) BTV-8 RNA in spleen samples taken 14 days after challenge was quantified using real-time RT-PCR targeting segment 5. The average segment 5 RNA copies per mg of spleen are shown. (D) BTV-8 virus titers in spleen samples were determined by end-point titration of supernatants on Vero cells and shown as average TCID_50_ per mg of spleen. Differences in virus titres between the groups were statistically evaluated with a Kruskal-Wallis rank sum test.

To evaluate the protective efficacy of the recombinant viruses against BTV-8 challenge, we utilized IFNAR^−/−^ mice as the infection model. After each injection, the applied dose of all vaccine inocula was confirmed by plaque assays on RK13 cells. In the first immunization, mice received 1.24×10^6^ plaque forming units (PFU) of rRacH1, 0.96×10^6^ PFU of rH_VP2 and 0.80×10^6^ PFU of rH_VP2_5. The booster doses were 1.25×10^6^ PFU, 1.05×10^6^ PFU, and 1.67×10^6^ PFU, respectively. Even though IFNAR^−/−^ mice are highly susceptible to a number of virus infections [26], all vaccinations were well tolerated and no adverse effects were observed in any of the mice. After challenge, all mock-vaccinated mice as well as mice that had received rRacH1 rapidly lost weight and died or had to be euthanized by day 6. The course of disease was slightly delayed in mice vaccinated with rH_VP2, but all mice in this group also died by day 7 after challenge infection (Fig. 4A&B). In contrast, mice in the rH_VP2_5 group only transiently displayed mild disease (stilted gait, ruffled coat) and weight loss (about 5%) by day 5, but fully recovered by day 9 (Fig. 4A&B). All mice vaccinated with the commercially available inactivated vaccine and the environmental controls stayed healthy and survived until the end of the experiment (Fig. 4A&B).

All spleens of mice that had succumbed to BTV-8 infection contained large amounts of viral RNA (over 1×10^7^ segment 5 copies per mg of spleen tissue). There was no significant difference in viral RNA levels between mice that succumbed to the infection in different groups (*P*>0.05). On average, spleens of mice vaccinated with rH_VP2_5 contained almost 50,000-fold less BTV-8 RNA than the spleens of mice vaccinated with rRacH1 (Fig. 4C). Only two of eight mice vaccinated with the inactivated vaccine were positive for BTV RNA.

In contrast, up to 10^6.8^ TCID_50_ of BTV-8 per mg of tissue were found in spleens of perished mice. On average, spleens of mice in the PBS group contained 10^5.6±1.1^ TCID_50_ per mg of tissue, compared to 10^5.1±0.6^ in the rRacH1 group, and 10^3.6±1.4^ in the rH_VP2 group. The difference between the rH_VP2 group and the other two groups was statistically significant (*P*<0.05) (Fig. 4D). No infectious virus could be re-isolated from the spleens of mice that had been vaccinated with rH_VP2_5 or BTVPUR.

**Figure 5:**
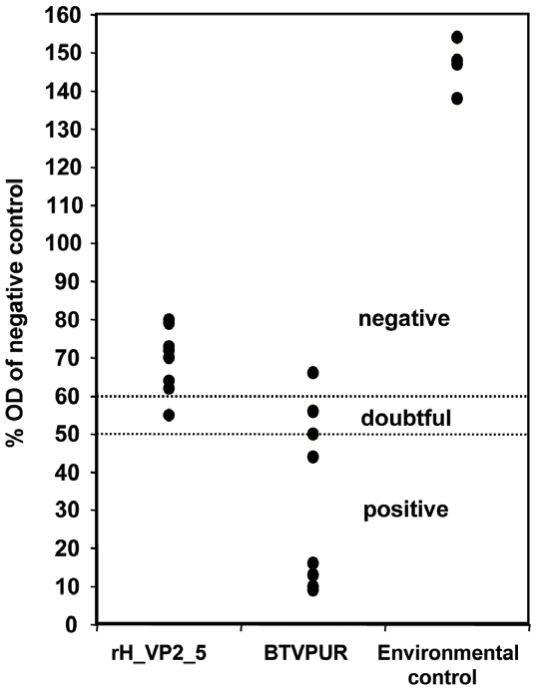
Detection of VP7 antibody from survived mice using ELISA. At the end of the experiment, antibody levels to BTV-8 VP7 were determined from blood samples of the surviving mice (rH_VP2_5, BTVPUR AlSap™ 8, and the environmental groups) with the ID Screen BT Competition ELISA. The OD values of samples were evaluated by comparing them to the kit negative control. All samples with an OD of up to 50% of the negative control are antibody-positive. Samples with higher ODs are considered doubtful (50% to 60%) or negative (over 60%).

Serum samples from surviving mice were tested in a VP7-specific ELISA, but blood samples of mice succumbing to infection were not available. All environmental controls stayed completely negative in the ELISA. Mice that had been vaccinated with rH_VP2_5 showed a clear, albeit weak, reaction in the test upon challenge infection. Seven of eight, however, remained on the negative side of the cut-off, compared to only one in the BTVPUR group (Fig. 5).

Taken together, our data suggest that the EHV-1 recombinant virus expressing VP2 and VP5 in combination but not VP2 alone can protect against BTV-8 challenge and that the recombinant vectored vaccine can be used as a DIVA when combined with a commercial VP7-specific ELISA.

## Discussion

Different vaccine strategies against BTV have been previously developed, including inactivated whole virus preparations, MLV vaccines, virus-like particles (VLPs) and vectored vaccines, of which only inactivated and MLV vaccines have been commercialized [7]. Both inactivated and MLV vaccines are highly efficacious in inducing long-term protection in ruminants [3], [30]. For inactivated vaccines, however, the cost of production is high and repeated immunization is needed; for MLV vaccines, the risk of causing adverse reactions in vaccinated ruminants and the potential reassortment with circulating wild-type viruses are of concern. The main argument against the use of either standard inactivated or MLV vaccines is, however, that they are not DIVA vaccines [3].

In contrast to the traditional inactivated or MLV vaccines, a DIVA strategy can be achieved using VLPs or live-vectored vaccines. VLPs are self-assembling structural viral proteins without BTV nucleic acids, which are safe as a reversion to virulence or genomic reassortment is impossible. Multivalent BTV vaccines are also possible when VLPs are administered as a mixture [31]. High production costs and stability issues with the assembled particles, however, have so far prevented commercialization of this approach.

On the other hand, vectored vaccines based on well-established poxvirus delivery systems have been developed. Since VP2 is an immunodominant protein harboring the most important neutralizing epitopes, it is usually the first candidate to be included in the vectored vaccines, alone or in combination with other proteins. A recombinant canarypox virus vectored vaccine co-expressing VP2 and VP5 of BTV-17 was shown to induce a sterilizing immune response in sheep [12]. With a replication-competent capripox virus encoding for VP2, VP7, NS1 and NS3 of BTV-2 individually, only partial protection was observed [11]. Based on these findings, we chose VP2, alone or in combination with its partner in the virus capsid, VP5, as transgenes expressed by recombinant EHV-1.

The potential of EHV-1 as a universal vector for immunization has been previously evaluated. It was shown to be very efficient in non-equine animals, mainly due to its capacity to deliver foreign genes in cells of various species and the lack of pre-existing anti-EHV-1 immunity [18]. EHV-1 has been used to deliver bovine viral diarrhea virus (BVDV) structural proteins in cattle and was shown to induce neutralizing antibodies that were correlated with reduced viremia and virus shedding [24]. While immunization of cattle with the recombinant EHV-1 induced neutralizing antibodies against EHV-1 as well, no cross-reactivity with bovine herpesvirus type 1 (BHV-1), a major pathogen of cattle, was observed [24]. This is of critical importance if a DIVA compatibility for BHV-1 is also needed. On the other hand, the lack of pre-existing anti-EHV-1 immunity in non-equine ruminants will avoid interference with the vector itself. This might be an issue with other vector systems such as capripox virus [11] and bovine herpesvirus type 4 (BHV-4) [32] that naturally occur in ruminants.

The limitations of BTV vaccine trials in natural hosts, such as the high cost for large animals, the need for large animal facilities of biosafety level 3 and the paucity of knowledge about their immune systems, has been overcome recently by the establishment of a small animal model, IFNAR^−/−^ mice [25]. Due to a deficiency in the β subunit of the IFN-α/β receptor, the mice are highly susceptible to many virus infections but can be protected by immunization [26], making them a versatile tool to evaluate the immune response and protection conferred by vaccination. Recently, IFNAR^−/−^ mice have been used to test BTV vectored vaccines based on recombinant modified vaccinia virus (MVA) and BHV-4 [32], [33].

We found that recombinant RacH co-expressing VP2 and VP5 of BTV-8 protected IFNAR^−/−^ mice against a lethal challenge infection. The mice displayed only transient and mild signs of discomfort, but fully recovered by day 9 after infection. No infectious virus was found in the mice, and viral RNA loads were dramatically reduced. With VP2 alone, the course of disease was slightly delayed, but all mice eventually succumbed to BTV-8 challenge. A similar result was reported when VP2 alone was expressed by a BHV-4 vector, even though the challenge dose in that experiment was much lower than the one used here [32]. These findings suggest that VP2 alone, while able to induce a neutralizing antibody response, can hardly provide complete protection against BTV infection. The degree of protection, however, is much greater when VP2 is used together with the minor outer capsid protein VP5 [13], [34]. This synergy may be derived from their close interaction in the virus particle, suggesting a strong conformational/folding dependence of VP2 on VP5. The presence of neutralizing epitopes not only in VP2 but also in VP5 has been postulated [4], but we did not test for antibodies to VP5 alone here.

The discriminatory potential of ELISAs based on non-structural proteins has already been demonstrated [35], [36], and those assays would be theoretically suited for a DIVA concept with existing inactivated vaccines. The recent BTV-8 vaccination campaigns, however, have shown that the practical value of a non-structural protein antibody ELISA is very limited, particularly in animals that have been vaccinated and revaccinated repeatedly. The carryover of non-structural proteins from culture system used to produce the vaccines often results in antibodies to viral non-structural proteins in vaccinated animals. While some of the inactivated vaccines in the market are highly purified, not all manufacturers include this cost-intensive step in their production process [9]. In consequence, attempts at establishing a commercial NS1 ELISA were not successful. Vaccination with inactivated vaccines from different companies led to an increased number of unspecific results in the test, which, consequently, was not released by the manufacturer.

If adequate protection can be achieved without using VP7, the well-established VP7 antibody ELISAs that are routinely used for BTV diagnosis can be used for DIVA. We demonstrate that the recombinant EHV-1 carrying both VP2 and VP5 protects mice against lethal BTV-8 challenge. While the highly sensitive real-time RT-PCR assay detected low levels of viral RNA in mice immunized with rH_VP2_5, no infectious virus was present. Compared to mice vaccinated with a standard inactivated vaccine, the rH_VP2_5 mice displayed only minimal levels of VP7 antibody even after challenge.

Together with the availability of infectious clones of EHV-1 vaccine strain RacH, which allows rapid generation of transgene-expressing vaccines, an approach based on vectored vaccines is a valuable alternative for protection against orbivirus infections, especially when a DIVA regimen is required.

## Materials and Methods

### Ethics statement

This study was carried out in strict accordance with German legislation on animal protection (Tierschutzgesetz). The experimental procedures were approved by the respective Ethics Committees in the federal states of Berlin (Permit No. LAGESO I C 112 – 0424/08) and Mecklenburg-Vorpommern (Permit No. LALLF M-V TSD/7221.3-1.1-058/10). The animal care facilities and programs of Freie Universität Berlin and the Friedrich-Loeffler-Institut meet all legal requirements. The experiments were carried out as approved by the Ethics Committees and all efforts were made to minimize suffering.

### Cells and viruses

Rabbit kidney (RK13) cells [18], [19], [20] and Vero cells (RIE0015, Collection of Cell Lines in Veterinary Medicine, FLI; derived from CCL 81, ATCC, Manassas, VA, USA) were maintained in Earle's minimum essential medium (EMEM) supplemented with 10% heat-inactivated fetal bovine serum, 100 U penicillin ml^−1^ and 0.1 mg streptomycin ml^−1^. EHV-1 parental viruses HΔgp2 (EHV-1 strain RacH in which gp2-encoding gene71 was replaced with a mini-F sequence) [20], rRacH1 (HΔgp2 in which gene 71 was restored), rH_VP2 (recombinant RacH expressing VP2 of BTV-8) and rH_VP2_5 (recombinant RacH expressing both VP2 and VP5 of BTV-8) were propagated and titrated in RK13 cells. The BTV-8 strain used in this study was a 2008 German isolate passaged two times on Vero cells [37].

### Plasmids and BAC mutagenesis

The complete VP2 and VP5 genes of BTV-8 were commercially synthesized after codon optimization (Genscript) and cloned as pUC57-BTV2 and pUC57-BTV5. The VP2 gene was then PCR-amplified using Finnzymes' Phusion high-fidelity DNA polymerase (New England BioLabs) with two oligonucleotides VP2-F/VP2-R (Table 1) and cloned into the *Bam*HI and *Xba*I sites of pEP-CMV-in to generate pEP-VP2. pEP-CMV-in was previously constructed from pcDNA3 (Invitrogen) by inserting the kanamycin resistance gene *aph*AI and a 18 bp *I-Sce*I restriction site flanked with two 50-bp duplicated sequences into the cytomegalovirus (CMV) promoter [38]. Two fragments (161 bp and 226 bp in length) flanked with *I-Ceu*I restriction sites were amplified from either side of the ORF1/2 deletion region in the RacH genome and cloned into pUC19 to generate pUC19-ORF1/2. The VP2-expressing cassette was released from pEP-VP2 by digestion with *Spe*I and *Sph*I, and cloned into the *Spe*I/*Sph*I sites of pUC-ORF1/2 to generate transfer plasmid pUC19-ORF1/2-VP2. By digesting with *I-Ceu*I, the VP2-expressing cassette with two flanking fragments was released from pUC19-ORF1/2-VP2 and used for the later recombination. To construct a VP5 transfer plasmid, an IRES sequence was amplified and cloned into the *Eco*RI and *Xba*I sites of pUC57-BTV5, resulting in plasmid pUC57-IRES-VP5. The kanamycin resistance gene *aphAI* and an *I-Sce*I restriction site with two flanks of 40-bp each were inserted using the *Xba*I site of pUC57-IRES-VP5 to generate transfer plasmid pUC57-IRES-kan-VP5.

**Table 1:** Oligonucleotides used in this study.

Primers	Sequences (5′ to 3′)
VP2-F	CGCGGATCC ATGGAGGAGCTGGCTATCCCC
VP2-R	GCTCTAGA TTACACGTTCAGAAGCTTCGTAAGC
ORF1 Fw	CAGTGAATTCGACGTAACTATAACGGTCCTAAGGTAGCGAATTTTTCCATTGGGCCCCTCCC
ORF1 Rv	CGCCTGCAGCTACTAGT TGGAGATGGAGACAGAGGAGG
ORF3 Fw	GATC GCATGC CCCGGGGCTAAAAAGCTGCGT
ORF3 Rv	GATCAAGCTGACGTAACTATAACGGTCCTAAGGTAGCGAAGGAGCAGCAGGCCCCCATCGA
EF1-ep1	TTTTGCGCACGGTTATGTGGACAAAATACCTGGTTACCCAGGCCGTGCCGGCACGTTAACCGGGCTCGTGAGGCTCCGGTGCCCGTCA
EF1-ep2	TGGTGGCGACCGGTAGCGCTAGCGGATCTGACGGTTCACTAAACCAGCTCTGCTTATATAGACCTCTCACGACACCTGAAATGGAAGA
IRES Fw	CAGT GAATTC GCCCCTCTCCCTCCCCCCCCCCT
IRES Rv	ATGC TCTAGA ATTATCATCGTGTTTTTCAAAGGAA
VP5-in Fw	ATGCTCTAGATCCAATATGGGCAAAATCATTAAGAGCCTGTCCCGTTGGATGACGACGATAAGTAGGGATAAC
VP5-in Rv	ATGC TCTAGA GGG TAA TGC CAG TGT TAC AAC CA
VP5-ep1	CATGTCTTTGGTAACGATGAGATGCTTACGAAGCTTCTGAACGTGTAAGCCCCTCTCCCTCCCCCCCCCCT
VP5-ep2	GCGAGCTCTAGCATTTAGGTGACACTATAGAATAGGGCCCTCTAGATCAGGCATTGCGAAGAAACAATGGG
pRacH-xx-F	CCCTCTACGGTTTTCTTCGAGGCCG
pRacH-xx-R	CCTAGGCGATGTGTGCAGCCGAGGC

The EHV-1 BAC pRacH1, derived from vaccine strain RacH, was constructed to harbor an inversion of the mini-F sequence relative to the parental clone pRacH described elsewhere [20] to increase genomic stability [19]. Here, pRacH1 was further modified by replacing the HCMV promoter upstream of *egfp* in the mini-F cassette with human elongation factor promoter 1α (EF-1α) to avoid potential recombination with the HCMV promoter present in the transfer plasmids. BAC mutagenesis was conducted using a two-step Red-mediated (*en passant*) strategy [38]. Briefly, the EF-1α promoter with the *I-Sce*I-*aphAI* cassette was amplified from pEP-EF1-in using oligonucleotides EF1-ep1/EF1-ep2 (Table 1), which carry sequences homologous to those flanking the HCMV promoter within mini-F sequences present in pRacH1. The PCR product was gel-purified and electroporated into *E. coli* GS1783 [39] cells harboring pRacH1. After the first recombination, the EF-1α promoter and *I-Sce*I-*aphAI* cassette were inserted into pRacH1, and kanamycin-resistant intermediates were obtained. For the second recombination, 1% arabinose was used to induce expression of the homing endonuclease *I-Sce*I, resulting in the cleavage of the *I-Sce*I restriction site upstream of the *aphAI* gene and, ultimately, the excision of the kanamycin cassette. The modified BAC was termed pH1_EF1. With the same strategy, the VP2 expression cassette was inserted in lieu of ORF1/2 of pH1_EF1 through recombination between BAC DNA and the fragment released by digestion with *I-Ceu*I from pUC19-ORF1/2-VP2, resulting in pH1_EF1_VP2. To insert the VP5 gene into pH1_EF1_VP2, two oligonucleotides VP5-ep1/VP5-ep2 (Table 1) were used to amplify IRES-VP5 and the *I-Sce*I-*aphAI* cassette from pUC57-IRES-kan-VP5. With another round of *en passant* recombination, the amplicon was inserted into pH1_EF1_VP2 utilizing 40-bp homology flanks present in the primers. The resulting pH1_EF1_VP2_5 BAC contained the VP2 and VP5 genes of BTV-8 separated by the IRES sequence and controlled by the common upstream HCMV IE promoter.

The strategy for the BAC mutagenesis is illustrated in Fig. 1. The correct construction of BAC mutants was assessed by restriction fragment length polymorphism (RFLP) analysis and sequencing. Virus reconstitution was performed by transfecting 1 µg of BAC DNA into RK13 cells using polyethylenimine (PEI) (Polysciences). gp2 expression of the reconstituted viruses was restored by co-transfection of 1 µg BAC DNA and 10 µg plasmid p71 in RK13 cells. The recombinant EHV-1 viruses expressing VP2 alone or VP2 and VP5 were named rH_VP2 and rH_VP2_5, respectively.

### Indirect immunofluorescence assays (IFA) and western blotting

For IFA, confluent RK13 cells in a 6-well plate were infected with rH_VP2, rH_VP2_5 or parental virus rRacH1 at a multiplicity of infection (m.o.i.) of 0.0001. One hour post infection (p.i), viruses were removed and infected cells were overlaid with 1.5% methylcellulose (Sigma) in EMEM-2% FBS. After 48 h of incubation at 37°C, cells were washed with 1× phosphate-buffered saline (1×PBS, 137 mM NaCl, 2.7 mM KCl, 4.3 mM Na_2_HPO_4_, 1.47 mM KH_2_PO_4_) and fixed in 3.5% paraformaldehyde in PBS for 30 min at room temperature (RT), followed by a 5 min incubation in PBS containing 30 mM glycine and another 5 min for permeabilization using PBS with 0.1% Triton X-100. After washing with PBS, cells were blocked with PBS-3% bovine serum albumin (BSA) for 30 min at RT and then incubated with monoclonal antibody (mAb) 13C10 against BTV-8 VP2 (a gift from Dr. Malte Dauber, Friedrich-Loeffler-Institut, Insel Riems, Germany) or 3B12 against EHV-1 gp2 [40] for 1 h at RT. After extensive washing (3 times for 10 min), the secondary antibody (Alexa Fluor568-conjugated goat anti-mouse IgG, Invitrogen) was added at a dilution of 1∶2,000 in PBS-3% BSA and incubated for 1 h at RT. After washing, the fluorescence signal was inspected under an inverted fluorescence microscope and recorded with a digital camera (Axiovert 25 and Axiocam, Zeiss).

For western blot analyses, RK13 cells were infected with the recombinant or parental EHV-1 viruses and Vero cells with BTV-8 wild type virus. Twenty-four hours p.i., cells were collected and resuspended in radioimmunoprecipitation assay (RIPA) buffer (20 mM Tris, pH 7.5; 150 mM NaCl; 1% Nonidet P-40; 0.5% sodium deoxycholate; 0.1%SDS) with a protease inhibitor cocktail (Roche) and benzonase (Novagen). Proteins of cell lysates were separated using sodium dodecyl sulfate (SDS)-12% polyacrylamide gel electrophoresis (PAGE) and transferred to a polyvinylidene fluoride (PVDF) membrane. After blocking the membrane with 5% non-fat dry milk in PBS, the membrane was incubated with mAb 13C10 against BTV-8 VP2, a sheep anti-BTV-8 hyperimmune serum [30] or mAb 3G4 against EHV-1 major capsid protein (MCP) in blocking buffer for 1 h. The secondary antibody was rabbit anti-mouse IgG-HRP (1∶1,000) (SouthernBiotech) or goat anti-sheep IgG conjugated with HRP (1∶1,000) (Santa Cruz Biotechnology). Reactive bands were visualized by enhanced chemoluminescence (Amersham ECL plus, GE healthcare).

### *In vitro* growth properties

To compare the *in vitro* growth properties of the recombinant viruses with EHV-1 parental virus, plaque areas and single-step growth kinetics were determined. Plaque areas were measured after infection of RK13 cells at an m.o.i. of 0.0001 and overlaid with 1.5% methylcellulose in EMEM–2% FBS. Three days p.i., IFA using mAb A8 against EHV-1 gM [20] was performed. For each virus, 50 plaques were photographed and mean plaque sizes were analyzed by using ImageJ software (http://rsb.info.nih.gov/ij/). For determining single-step growth kinetics, RK13 cells seeded in 24-well plates were infected at an m.o.i. of 5. The viruses were allowed to attach for 1 h at 4°C, followed by a penetration step of 1.5 h at 37°C. After washing twice with PBS, infected cells were treated with ice-cold citrate buffered saline (pH 3.0) for 3 min to remove residual virus. At different time points (0, 4, 8, 12, 24, 36, 48 h p.i.), supernatants and infected cells were collected separately. Extracellular and cell-associated virus titers were determined by plaque assays. Single-step growth curves were computed from three independent experiments.

### Immunization and challenge infection of mice

In the first animal experiment, we determined whether the recombinant viruses induced a BTV-8-specific humoral immune response. Three-week-old female Balb/c mice (Harlan) were allocated randomly to five groups of 6 mice each. Each mouse was primed and booster-immunized with 1×10^5^ plaque forming units (PFU) of virus in a 3-week interval. Group I was inoculated intranasally (IN) with rH_VP2. For Group II, the same virus was used, but the application was subcutaneous (SC). Group III and IV were vaccinated with rH_VP2_5 IN or SC, respectively. The mice in the control group were immunized with parental rRacH1. Blood was collected from mice 1 day before immunization (day-1) and on days 14, 21, 28, and 35. Serum was prepared by centrifugation at 5000 rpm for 2 min. The antibody titers were examined in a standard SNT [41].

In the second animal experiment, the protective efficacy of the vectored vaccines against BTV-8 challenge was evaluated using 36 male IFNAR^−/−^ mice (on a C57BL/6 genetic background), which were provided by Dr. Markus Keller, Friedrich-Loeffler-Institut, Insel Riems, Germany. Two groups of eight mice were inoculated with rH_VP2 or rH_VP2_5. Four mice received parental rRacH1. Eight mice served as positive vaccination controls and were injected with 50 µl of a commercially available inactivated BTV-8 vaccine (BTVPUR AlSap™ 8, Merial, Lyon, France; licensed for use in domestic ruminants in the European Union). A control group of four mice was mock-vaccinated with 100 µl of PBS. Another group of four mice was kept in the same room as the other mice, but was not vaccinated (environmental control). The immunizations were given SC twice three weeks apart. Three weeks after the second application, all mice except the environmental control group were challenged by subcutaneous injection of 5×10^3^ TCID_50_ of BTV-8 in cell culture medium. The intraperitoneal LD_50_ of this strain for IFNAR^−/−^ mice is about 10^−1^ TCID_50_ (M. Eschbaumer, unpublished observations). The challenge dose was confirmed by titration on Vero cells.

### Tissue sample preparation

Mice were weighed every morning, checked for signs of disease at least twice a day, and dead mice were removed from the cages immediately. Mice that met pre-defined exit criteria (apathy, ruffled fur and ocular discharge or over 20% weight loss) were anesthetized with isoflurane and killed by cervical dislocation. The whole spleen was removed from dead or euthanized animals, suspended in 1 ml of serum-free MEM containing antibiotics and antimycotics, weighed and stored at −70°C until analysis.

### RNA extraction and quantitative RT-PCR

For RNA extraction and virus titration, the samples were thawed and homogenized with steel balls in a TissueLyser (Qiagen, Hilden, Germany). Homogenized spleen samples were centrifuged, and the supernatants removed. One hundred microliters of supernatant were added to 300 µl of MagNA Pure LC lysis/binding buffer (Roche Diagnostics) and total nucleic acid was extracted using a MagNA Pure LC according to the manufacturer's instructions. BTV-8 RNA was detected by semi-quantitative RT-PCR (RT-qPCR). Of 100 µl of total nucleic acid eluate, 5 µl were used for RT-qPCR. Amplification was performed with the AgPath-ID™ One-Step RT-PCR kit (Applied Biosystems/Ambion) using a “pan-BTV” genome segment 5 PCR with primers and probes exactly as described [42]. In vitro transcribed BTV-8 segment 5 RNA standard (B. Hoffmann, unpublished; protocol available upon request) was used for absolute quantification. BTV-8 virus titers in spleen samples of mice were determined by end-point titration of supernatants on Vero cells. Differences in virus titers between groups were statistically evaluated with a Kruskal-Wallis rank sum test.

### Detection of VP7 antibody using ELISA

At the end of the experiment (two weeks after challenge infection), blood samples were taken from all surviving mice. Blood was collected in plain tubes. After coagulation, samples were centrifuged and sera were harvested and stored at −70°C until analysis. Antibody levels to BTV-8 core capsid protein VP7 were determined with the ID Screen BT Competition ELISA (ID Vet). The optical density values (ODs) of samples are evaluated by comparing them to the kit negative control. All samples with an OD of up to 50% of the negative control are positive. Samples with higher ODs are considered doubtful (50% to 60%) or negative (over 60%).
